# A protein-proximity screen reveals Ebola virus co-opts the mRNA decapping complex through the scaffold protein EDC4

**DOI:** 10.21203/rs.3.rs-3838220/v1

**Published:** 2024-02-02

**Authors:** Robert Davey, Callie Donahue, Aditi Kesari, Naveen Thakur, Ling Wang, Sarah Hulsey-Stubbs, Caroline Williams, Cara Kirby, Daisy Leung, Uma Aryal, Christopher Basler, Douglas LaCount

## Abstract

The interaction of host and Ebola virus (EBOV) proteins is required for establishing infection of the cell. To identify protein binding partners, a proximity-dependent protein interaction screen was performed for six EBOV proteins. Hits were computationally mapped onto a human protein-protein interactome and then annotated with viral proteins to reveal known and previously undescribed EBOV-host protein interactions and processes. Importantly, this approach efficiently arranged proteins into functional complexes associated with single viral proteins. Focused characterization of interactions between EBOV VP35 and the mRNA decapping complex demonstrated that VP35 binds the scaffold protein EDC4 through the C-terminal subdomain, with each protein found associated in EBOV-infected cells. Mechanistically, depletion of three components of the complex each similarly inhibited viral replication by reducing early viral RNA synthesis. Overall, we demonstrate successful identification of EBOV protein interaction with entire cellular machines, providing a deeper understanding of replication mechanism for therapeutic intervention.

### **Introduction:**

Ebola virus (EBOV) causes severe hemorrhagic fever and imposes a heavy public health burden with increasing outbreak frequency. Within the last decade, the two largest EBOV outbreaks have occurred in Western Africa and the Democratic Republic of the Congo, both marked by high fatality rates[1]. Several promising antibody-based medical countermeasures targeting the virus glycoprotein have recently been developed and approved for use in disease treatment, however these therapeutics must be administered early in infection to be effective[2]. A better understanding of virus replication mechanism may provide additional targets for drug development.

EBOV is a negative sense RNA virus with a genome that contains seven genes bordered by 3' and 5' leader and trailer sequences. RNA synthesis is driven by the viral polymerase L in conjunction with cofactors NP, VP35 and VP30, which together make up the RNA-dependent RNA polymerase (RdRp) complex. Viral transcription produces a gradient of mRNAs, with abundant transcripts from genes at the 3' end and a gradual decrease for genes closer to the 5' end[3]. Genomic replication initiates at the 3' leader sequence and initially synthesizes a positive-sense antigenomic RNA intermediate. Concurrent production of the negative-sense genomic strand initiates at the complement of the trailer region[3]. Both genomic replication and transcription occur in viral inclusion bodies formed by the accumulation of NP[4] that additionally contain VP35, VP30, L, and RNA[5]. EBOV virions consist of seven proteins, including the glycoprotein (GP), matrix protein VP40, the nucleocapsid proteins VP24, VP35 and NP, which coats the RNA genome, and the additional transcription and replication proteins VP30 and L[6]. Viral transcription and genomic replication initiate following virus entry and uncoating.

EBOV replication depends on the multifunctionality of its own proteins as well as virus-host protein interactions to successfully replicate and evade host antiviral responses. Through interactions with NP, VP35 influences the assembly of viral inclusion bodies[4,7]. VP35 caps the blunt end of viral dsRNA, preventing detection by RIG-I sensing and concurrent IFNβ production[8]. In addition to masking viral RNA detection, VP35 abrogates host cell antiviral responses by interacting with host proteins such as the kinases IKKε and TBK-1, preventing the synthesis of Type 1 interferons[9].

Recently, several groups have sought to identify EBOV-host protein-protein interactions (PPI) through interaction screening[10–16]. Each screen has provided large volumes of information regarding individual host protein-virus protein interactions, but host proteins are usually found in functional complexes, not in isolation[17]. To better identify host protein complexes interacting with virus proteins, we tagged each EBOV structural protein (except the transmembrane glycoprotein) with a promiscuous biotin ligase (BioID2) to label host interactors via proximity-dependent biotinylation[18]. Here, we show that computationally inter-relating the host-virus hits to known human protein-protein interaction (PPI) networks allows for the rapid identification of host protein complexes that interact with each virus protein, such as the mRNA decapping complex, which we characterize as an interaction partner of VP35. Our data demonstrates the power of this approach to identify cellular protein complexes necessary for productive infection.

### <u>Results</u>:

#### Proximity-dependent biotinylation screen identifies 447 host protein interactors of EBOV proteins

N- and C- terminal BioID2-tagged constructs were developed for NP, VP35, VP40, VP30, VP24 and L (**Figure 1a**). As a control to measure nonspecific biotinylation we included GFP fused to BioID2 as well as parental cells not expressing BioID2. Constructs were expressed in tetracycline inducible Flp-In T-Rex 293 cells to provide comparable levels of expression. Nonetheless, expression varied (for example between VP35 N and C terminal fusions), likely reflecting stability of the fusion protein (**Figure 1b**). Consistent with previous work[5,10], the RNA-dependent RNA polymerase, L, was expressed at the lowest levels, with the N-terminal BioID2 tagged version present at levels just above the limit of detection. The C-terminal fusion to L was not detected and was therefore excluded from further analysis.

Biotinylation of cellular proteins in close proximity to tagged viral proteins was activated by incubating cells with excess biotin for 24 hours. Cells were then lysed and biotinylated proteins purified on streptavidin beads. Five percent of the purified protein was subjected to western blotting to confirm successful biotinylation and protein capture **(Figure 1c**). The pattern and level of biotinylation was consistent between replicates but varied between constructs. While faintly expressed constructs yielded the lowest levels of biotinylated host proteins, others such as the N-terminal fusions to NP gave much higher levels compared to their C-terminal constructs (**Figure 1b-c, Extended data figure 1**). This may reflect mechanistic restrictions on the protein. For example, C-terminal fusions to VP35 can disrupt folding due to the positioning of the C-terminal end in the hydrophobic pocket of the IID region of the protein[8], which may result in protein instability.

The remainder of the samples were subjected to mass spectrometry to identify biotinylated proteins. Spectral counts for each protein were filtered by SAINTExpress against the CRAPome database to identify non-specifically labeled proteins and develop a set of high confidence interactors[19]. Using GFP-BioID2 and unmodified parental Flp-In T-Rex 293 cells as background and a Bayesian false discovery rate of 0.01 as the cutoff, 330 proteins were identified (**Extended data table 1**). Of these, fifty-two host proteins were identified by both N- and C- terminal BioID2 constructs of the same viral protein. Seventy-two cellular proteins interacted with multiple viral proteins (**Extended data table 1**), mainly due to overlap with NP-NT, which demonstrated the highest biotinylation activity of all constructs. Therefore, to identify cellular proteins that were specifically labelled by individual viral proteins, we repeated the SAINTExpress analysis for each viral protein using the mass spectrometry data from all other viral proteins plus GFP and parental cells as background. The resulting data set was significantly reduced in size and eliminated all but one shared interaction (**Extended data table 1**).

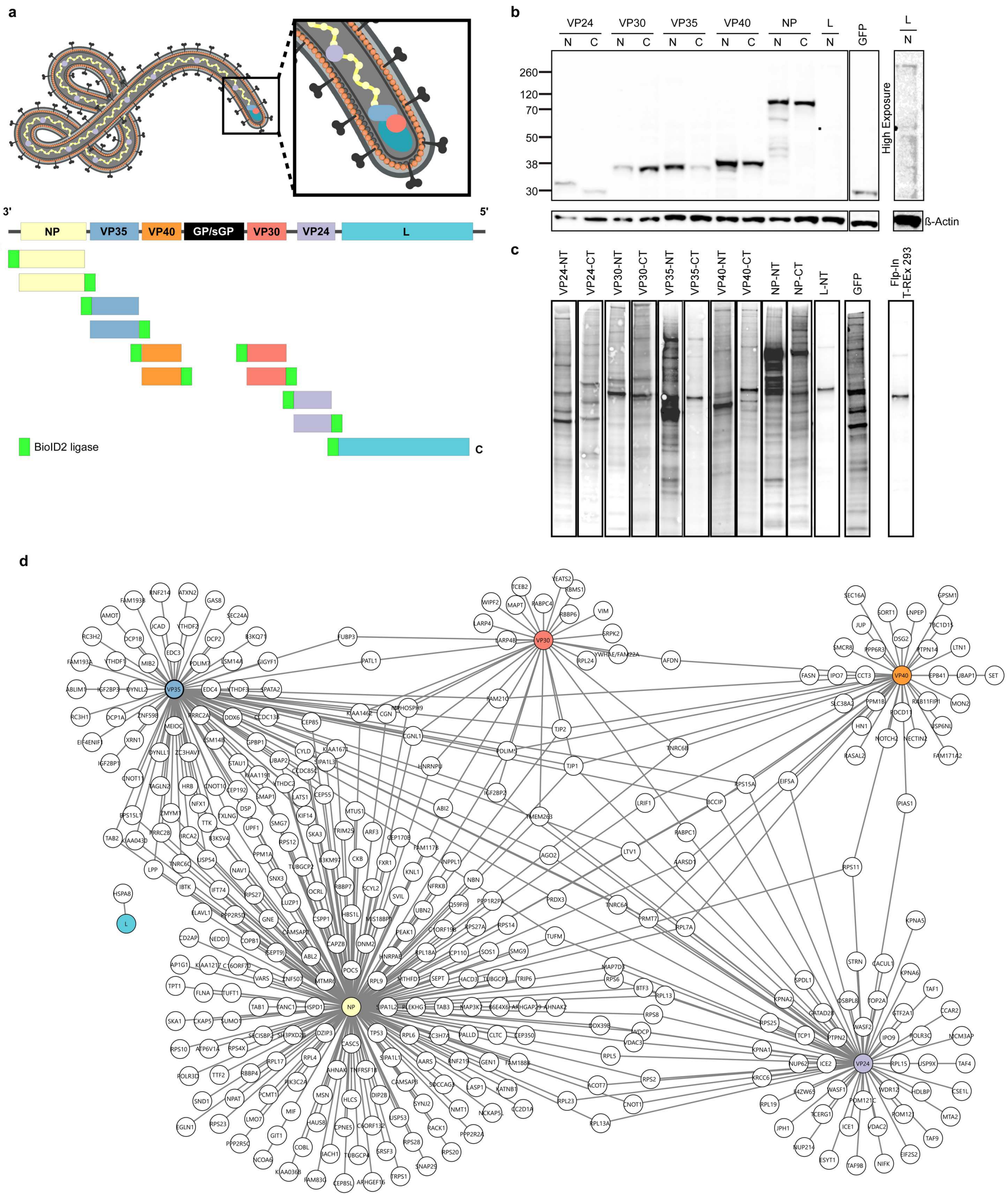

**Figure 1: A protein proximity screen for Ebola virus proteins identifies an expanded viral interactome**. **a** Top schematic shows arrangement of EBOV structural proteins in virion and encoding genes in the virus genome. Bottom shows constructs used in screening with placement of the BioID2 ligase indicated as green blocks. **b** Expression of viral protein-BioID2 fusion constructs and GFP-BioID2 non-specific control from stable cell line lysates. Blots were probed with biotin ligase specific antibody and loading levels controlled by detection of β-actin. **c** Blot of biotinylation levels, measured in cell lysates after inducing expression of each indicated protein by tetracycline and then adding biotin to cell medium to measure ligase activity. GFP-BioID2 was used as non-specific biotinylation control (second to last lane) as well as endogenous biotinylation in Flp-In TREx 293 cells not expressing any BioID2 construct (last lane). One representative experimental replicate of each construct is shown. **d** Hub and spoke network model showing identified interactions from screening. 447 host-viral protein interactions were identified between both N- and C- terminal constructs.

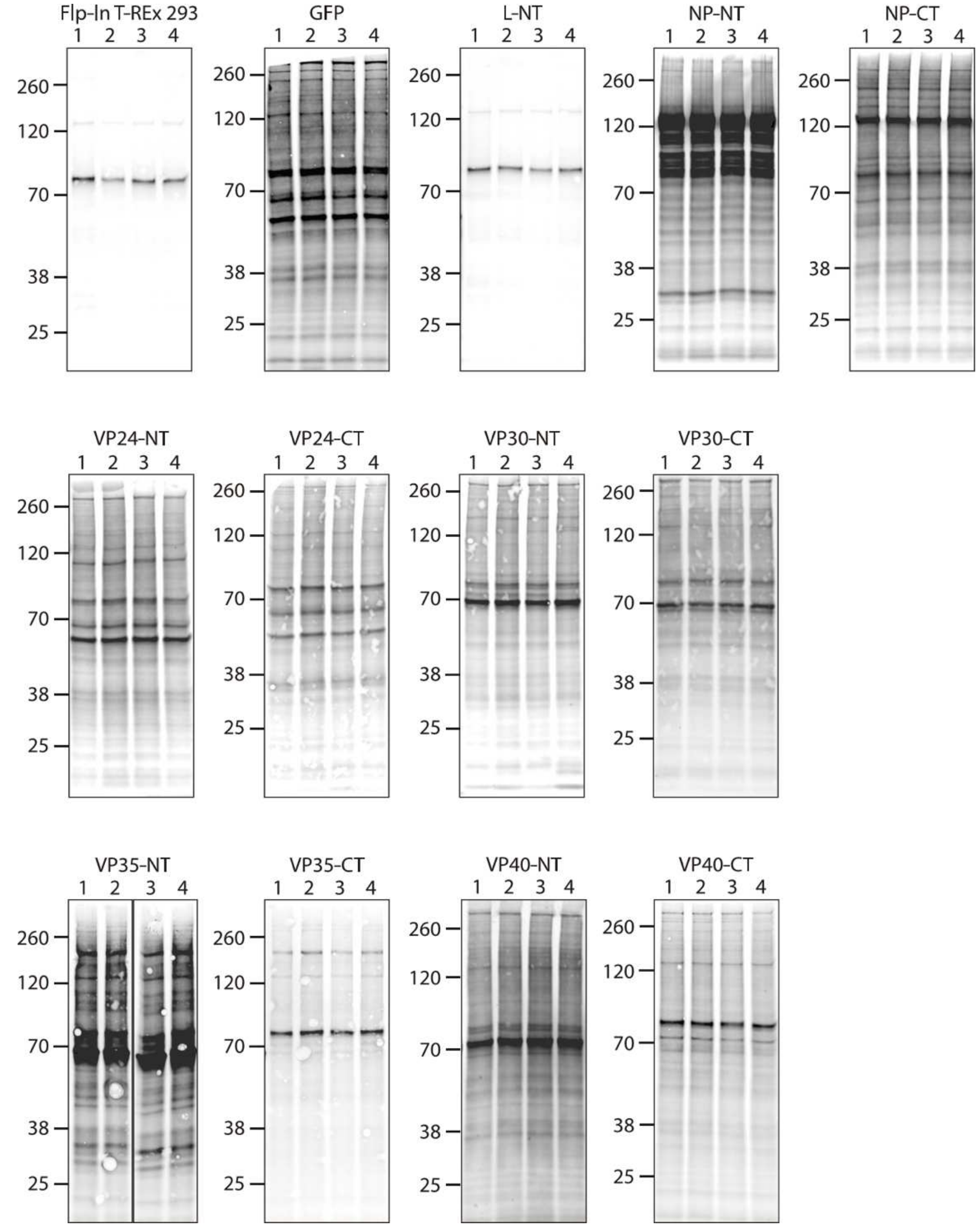

**Extended data figure 1: Viral BioID2 construct biotinylation abundances.** Each BioID2 screen was performed in quadruplicate. The top left shows endogenous biotinylation in the Flp-In TREx 293 cell line, which expresses no BioID2 construct, and the following panel represents non-specific biotinylation by the GFP-BioID2 construct. Subsequent panels demonstrate biotinylation of the indicated constructs. Following processing, samples were transferred to nitrocellulose membranes and stained with fluorescently labeled streptavidin.

**Network analysis and viral protein mapping reveals potential interactions of virus proteins with host protein complexes**

Combined hit lists for the BioID SAINTExpress analyses yielded 447 proteins of interest (**Extended data table 2**). As has been performed in other PPI screens, a hub and spoke network model was made showing the pairwise relationships of identified virus-host protein interactions (**Figure 1d**). However, many cellular proteins execute their functions as part of larger protein complexes and so we sought approaches to relate hits according to known host protein-protein interactions (PPI). Computationally inter-relating human PPI with the BioID hits using traditional methodologies such as STRING[20] yielded a highly interconnected mass of interactions involving 337 hits but lacked prioritization by hit score or complex likelihood (**Supplemental Figure 1**). As an alternative we implemented the Prize-Collecting Steiner Forest (PCSF) algorithm[21], a graphing methodology that limits protein connection paths using cost-based evaluation of connections to prioritize higher-confidence (low cost) interactions from the underlying human PPI and negatively weigh low-confidence (high cost) interactions (**Figure 2a**). As needed, PCSF also incorporates nodes from the underlying network, known as Steiner nodes, to form connections between hits to develop an optimal network (**Figure 2a**)[21].

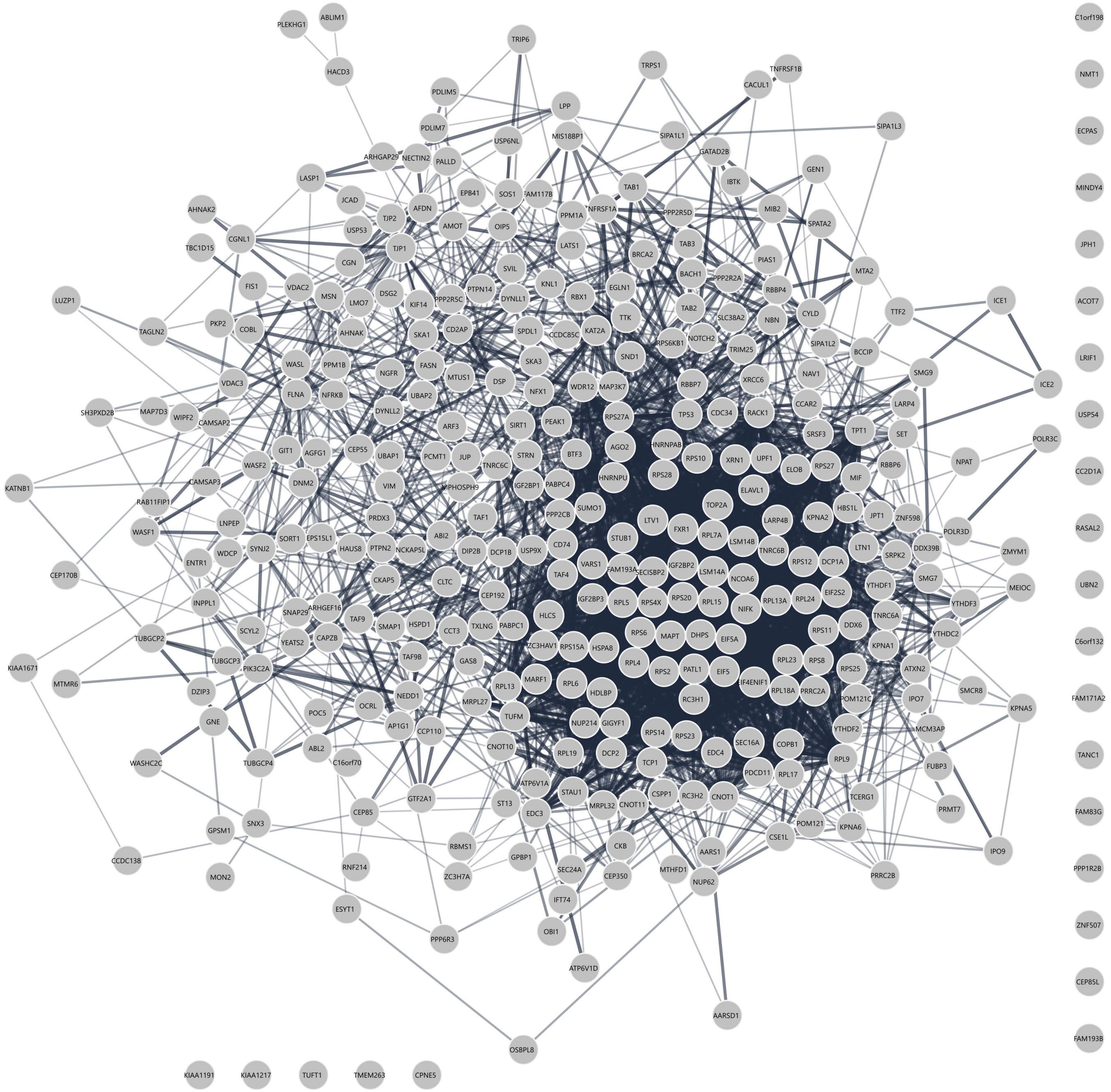

**Supplemental Figure 1: STRING network analysis of BioID hits.** STRING v12.0 was used to map BioID hits to the STRING database. The thickness of the black edge lines correlate to edge confidence scoring (0.4-1.0) while grey circles represent protein hits from the screen. Grey circles on the right side and bottom of the figure not connected by dark grey lines show hits within the STRING database that could not be connected to the final network.

SAINT scores from the viral BioID2 hits were used as node prize values (**Extended data table 2**) and hits were mapped onto the Human Integrated Protein-Protein Interaction rEference v2.3 (HIPPIE), an interactome assembled and scored from experimentally validated PPIs, in which interactions are given a confidence metric (edge weight) based on the number of publications and types of experimental approaches supporting the interaction[22]. 313 hits were mapped through PCSF. The virus proteins interacting with each host protein were then annotated onto the network (**Figure 2b left, Figure 2c**). While most interactions were between only one virus and host protein, 16% of hits were found associated with multiple viral proteins and are indicated via segmented circles with proportions defined by SAINT scores (**Figure 2b left**, **2c**, **Extended data table 3**). From this analysis, groups of host proteins related by a common virus protein(s) were apparent. To determine if these groups had functional relevance, the network was clustered based on network topology and gene list enrichment was performed on each cluster using EnrichR[23] (**Figure 2b right**). Interestingly, clusters related by common virus proteins (**Figure 2c**) showed strong enrichment (adjusted p-values 0.014 to $8.36 * 10^{-19}$ ) for functional pathways and were annotated as an additional layer onto the network (**Figure 2d, Extended data table 4**).

The PCSF subnetwork clusters included known EBOV-host protein interactions but revealed new associations with functionally related host proteins. For example, VP24 was associated with multiple karyopherin (KPNA) proteins[24] (**Figure 2c**) but also with related components of the nuclear pore complex not previously described for relevance in EBOV infection, such as CSE1L, which promotes the nuclear export of karyopherins[25] (**Figure 2d, cluster 16, Extended data table 4**). Additionally, markers of cell-signaling processes known to be relevant in EBOV infection were seen but with novel EBOV interactors. Rho-GTPase signaling is necessary for both EBOV entry and assembly[26], but was found represented by a cluster of proteins associated with NP and VP24, which extends previous findings that actin polymerization is necessary to drive transport of mature viral nucleocapsids to sites of viral budding[27](**Figure 2d, cluster 3, Extended data table 4**). Also, while the viral glycoprotein was previously found to induce NFκB signaling[28], our analysis suggests NFκB signaling may also correlate to a cluster of NP and VP35 associated host proteins, suggesting additional viral mediators to this host signaling pathway (**Figure 2d, cluster 15, Extended data table 4**).

We focused on a cluster of five proteins, DCP1A, DCP1B, DCP2, EDC3, and EDC4, labeled by VP35 (**Figure 2c inset**). This cluster was enriched for mRNA decay by 5' to 3' exoribonuclease as well as processing-body (P-body) formation (**Figure 2d**, **cluster 10, Extended data table 4**). Consistent with the functional enrichment, all five proteins are components of the host mRNA decapping complex[29]. Since biotinylation can involve direct as well as indirect interactions within 10 nm of the BioID2 enzyme[18] we predicted this cluster of proteins represented VP35 interaction with an intact host protein complex.

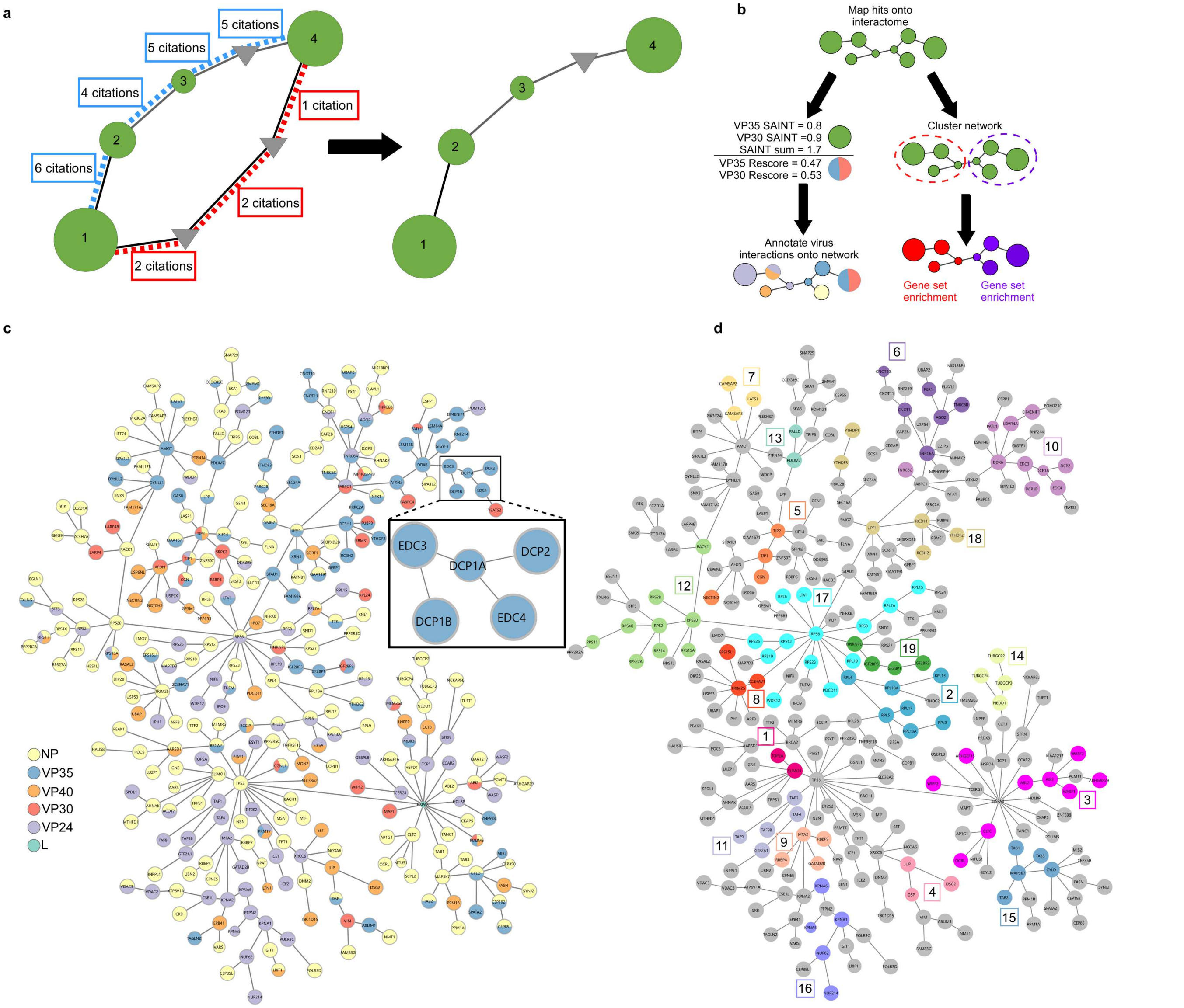

**Figure 2: Network analysis reveals virus interactions with putative host protein complexes.** PCSF was used to inter-relate BioID hits with the HIPPIE human protein-protein interaction (PPI) network. **a** The algorithm seeks to connect hits via the shortest pathways defined by the PPI network and considers confidence of underlying interactions and the presence of other hits along the mapped path. Hits are represented by nodes (green circles), where node size corresponds to the BioID hit score. At left, two paths are identified to connect strongly scored hits 1 and 4. The red path is the shortest to make the connection but requires inclusion of two host proteins that were not hits (Steiner nodes, triangles). The blue route is longer but has higher confidence, being better supported by literature citations, incorporates other hits from the screen and only one Steiner node. The algorithm will have preference for incorporating the blue route into the final network solution at right. **b** Overview of complete network mapping and gene set enrichment process. (Left) indicates how virus protein interactions from BioID screening were rescored as a portion of total SAINT scores for an associated host protein interaction. If a host protein was associated with multiple virus proteins it was labeled by a segmented circle using colors indicated in c (Right) to perform functional enrichment, subnetwork clusters were segregated by edge-betweenness measurements (red and purple dashed circles indicate clusters). Gene set enrichment was then performed for each cluster (red and purple circles). **c** Full network generated by PCSF for identified BioID2 screening hits related by the HIPPIE PPI. Five proteins of the mRNA decapping complex, each bound by VP35, are indicated in the inset. **d** Gene ontology annotated network obtained by Enrichr. Each color and corresponding number designate a different subnetwork with a functional enrichment listed in Extended data table 2.

**EBOV VP35 interacts with the host mRNA decapping complex scaffold protein EDC4**

To identify interactions between VP35 and each member of the decapping complex, co-immunoprecipitations of exogenously expressed proteins were performed from lysates of HEK293T cells transfected with plasmids encoding FLAG-tagged VP35 together with HA-tagged DCP1A, DCP1B, DCP2, EDC3 or EDC4. Under the conditions tested, VP35 only coprecipitated with EDC4 (**Figure 3a, last lane**). This outcome was consistent with spectral labeling counts from the BioID screen, showing EDC4 was the most abundantly labeled decapping complex component in the VP35 BioID (**Extended figure 2**). To confirm the EDC4-VP35 interaction, tags were reversed with plasmids encoding FLAG-tagged EDC4 and HA-tagged VP35 and pulldowns were performed with both anti-FLAG and HA antibodies (**Figure 3b, top and bottom panels respectively**). Our results suggest that VP35 interacts with the decapping complex by binding EDC4.

To confirm interaction of EBOV VP35 and EDC4 in infected cells, each protein was stained with specific antibodies at 10 and 20 hours post infection (hpi) with infectious EBOV. For EDC4, staining was present in distinct puncta in the cytoplasm. Interestingly, for infected cells, 20-40% of the puncta were found localized with inclusion bodies identified by VP35 staining (**Figure 3c-d**). To gain further insight into this association, z-stack images were collected from infected cells stained for VP35 and EDC4. Images were deconvolved and modeled using Imaris imaging software. EDC4 was confirmed to be associated with VP35 both within and proximal to VP35 inclusions (**Figure 3d, Supplemental movie 1**). Also, a low level of staining for EDC4 and VP35 in small puncta distinct from viral inclusion bodies was evident. To determine if this population of proteins was interacting, proximity-ligation assays were performed to detect proteins within 40 nm of each other. Despite some background signal, infected cells showed both puncta and diffuse patches of signal that was distinct from that seen in uninfected cells (**Figure 3e**). Taken together these findings indicate that during EBOV infection VP35 and EDC4 interact with one another.

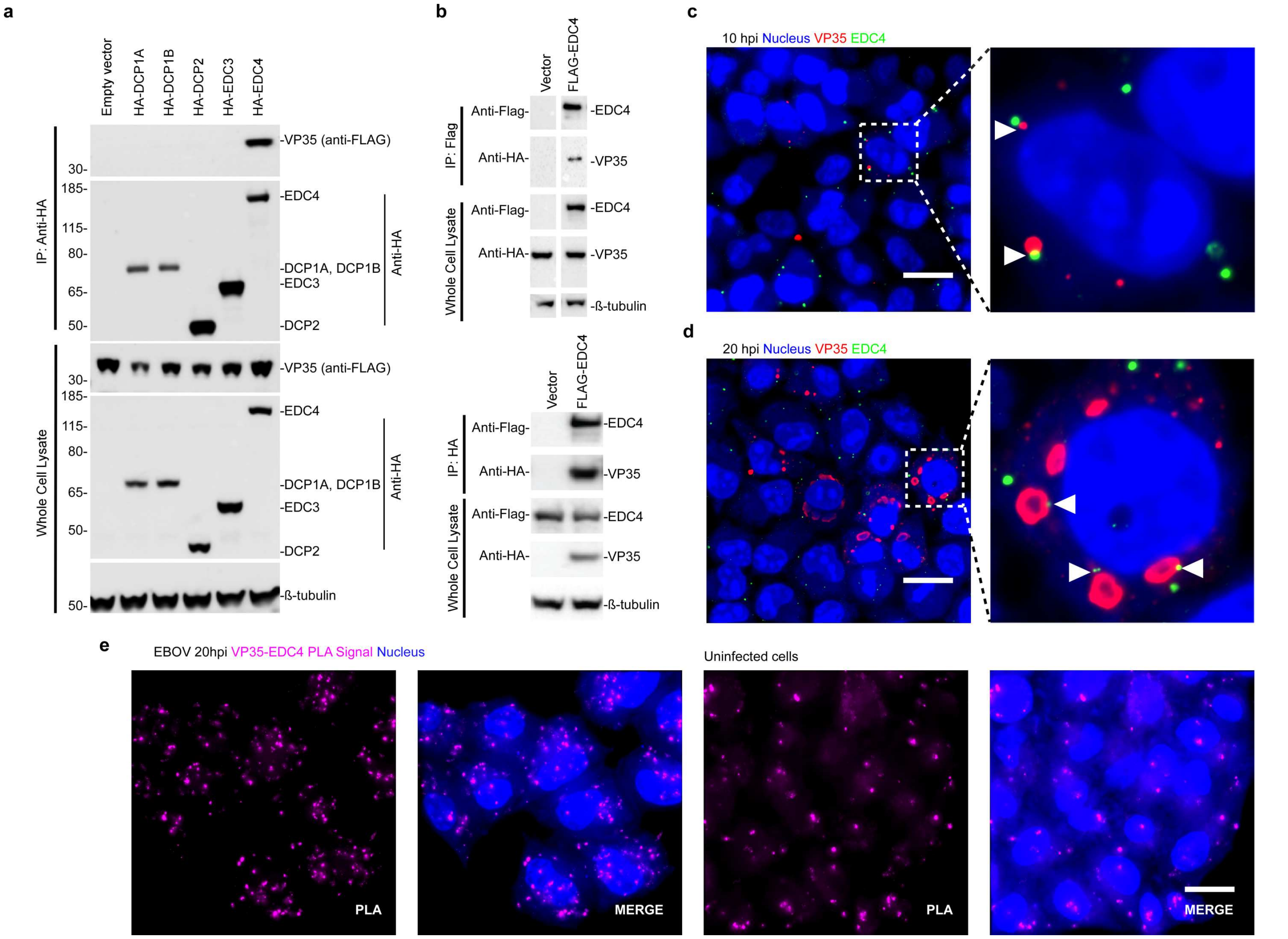

**Figure 3: VP35 interacts with the decapping complex through EDC4.** **a** Pulldowns of HA-tagged mRNA decapping proteins using anti-HA beads were performed in the presence of Flag-tagged VP35. Under the conditions, VP35 only coprecipitated with EDC4 (lane 5). **b** Confirmation of the VP35-EDC4 interaction. Pulldowns were performed with Flag-tagged EDC4 using anti-Flag beads (top panel) or HA-tagged VP35 using anti-HA beads (bottom panel). **c-d** EDC4 proximally localizes to VP35 inclusions during infection. Cells were challenged with EBOV at an MOI of 1 and fixed **c** 10 hpi and **d** 20 hpi. EDC4 puncta were identified colocalizing with VP35 at the edges of viral inclusion bodies (triangles). Scale bar = 25 µM. **e** Populations of EDC4 and VP35 colocalize during infection. Cells were challenged with EBOV and fixed 20hpi. Proximity ligation analysis was performed with antibodies directed towards EDC4 and VP35. Signal was observed in infected cells (left) over uninfected cells (right). Scale bar = 25 uM. One representative experimental replicate is shown.

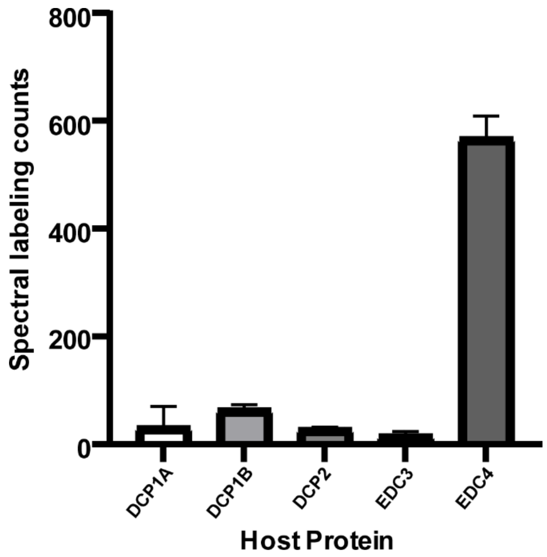

**Extended data Figure 2: EDC4 is abundantly labeled by VP35 BioID2.** Spectral labeling counts for each decapping protein showed that EDC4 was the most abundantly labeled protein of all decapping complex proteins identified.

**EDC4 regulates EBOV replication at an early step in viral infection**

Since EDC4 regulates mRNA stability through the decapping complex, we hypothesized that EDC4 depletion would effect virus RNA (vRNA) replication. Therefore, to understand the importance of the VP35-EDC4 interaction to the viral infection cycle, we depleted EDC4 protein with multiple siRNAs targeting different regions of the EDC4 gene in HeLa cells and challenged treated cells with EBOV. The amounts and subcellular localization of vRNA was then visualized by RNAFISH. Depletion of EDC4 (>80% for each siRNA, **Figure 4a**) resulted in 56% and 84% loss of cells with vRNA signal over multiple experiments (p-value < 0.01) compared to non-targeting (Allstars) siRNA controls, confirming that EDC4 plays a significant role in viral replication (**Figure 4b-c**). To determine which steps in viral replication were affected we measured both EBOV mRNA and genomic RNA levels in cells treated with siRNA. Oligo-dT reverse transcription (RT) combined with qPCR was used to specifically amplify poly-adenylated RNAs and measure NP and GP transcripts. In cells depleted of EDC4 by each siRNA, NP transcript levels were seven and fourteen-fold reduced compared to non-targeting siRNA controls (**Figure 4d,**

**first panel**) while GP transcript levels were 3 and 6-fold reduced compared to non-targeting controls **(Figure 4d, second panel).** The reduced mRNA levels appeared to result in a proportional 2 to 4-fold loss of viral protein levels, demonstrated by measuring VP35 protein levels (**Supplemental figure 2**). Next, levels of viral genomic and antigenomic RNA were measured in cells depleted for EDC4 by a previously described qPCR assay using primers specific for the virus trailer region[30]. Genomic RNA production was decreased 3 to 5-fold in EDC4 knockdown samples when compared to controls (**Figure 4d, third panel**), while antigenomic RNA production was decreased four to six-fold in EDC4 knockdown samples **(Figure 4d, last panel),** indicating that EDC4 is required for efficient viral RNA synthesis.

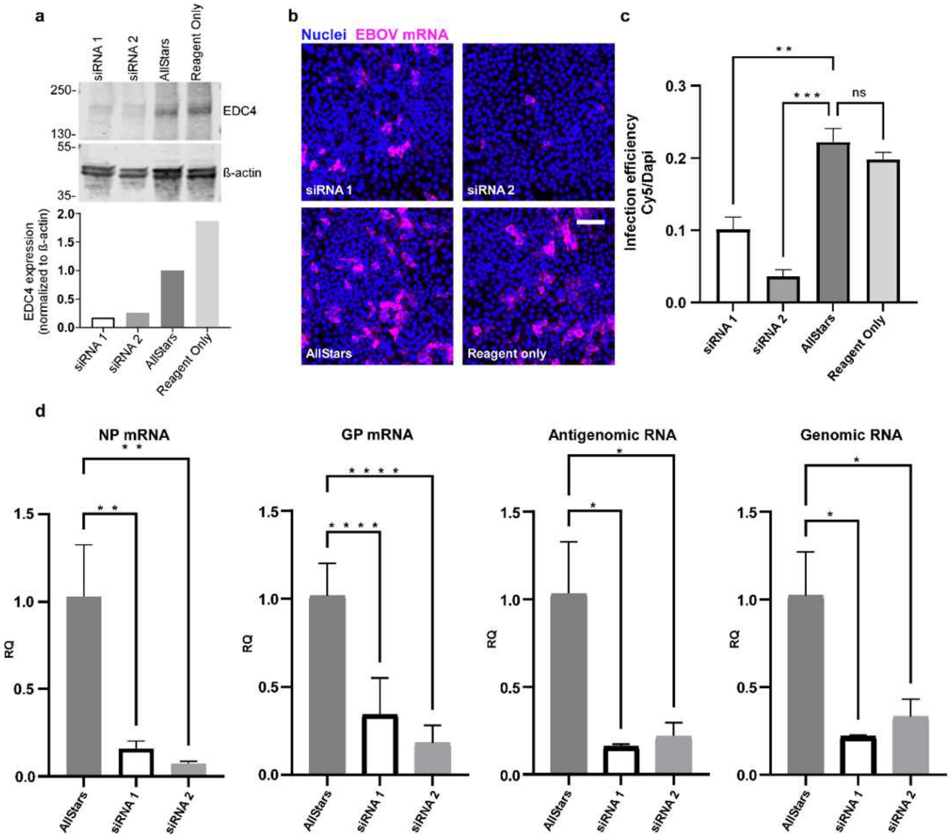

**Figure 4: EDC4 levels impact viral RNA production. a** siRNA depletion of EDC4 protein levels measured by immunoblot probed with specific antibodies. Lower panel shows quantitation of a representative replicate of three independent experiments measured by area under the curve and normalized to β-actin expression levels. **b** Viral replication was measured by RNAFISH staining using multiple oligonucleotides complementary to EBOV NP and VP35 mRNA. Scale bar = 100 µM. **c** Quantification of 10 images for which panel **b** is representative. P-values ** <0.01; *** <0.005. **d** EDC4 depletion results in reduction of viral mRNA and genomic RNA synthesis. Each indicated set of RNA transcripts were quantified via qPCR. Antigenomic RNA indicates positive sense RNA and genomic RNA is negative sense RNA. Where applicable, statistical significance was calculated by ANOVA. P-values were * <0.05; ** <0.01; **** < 0.0001. One representative experimental replicate is shown per qPCR.

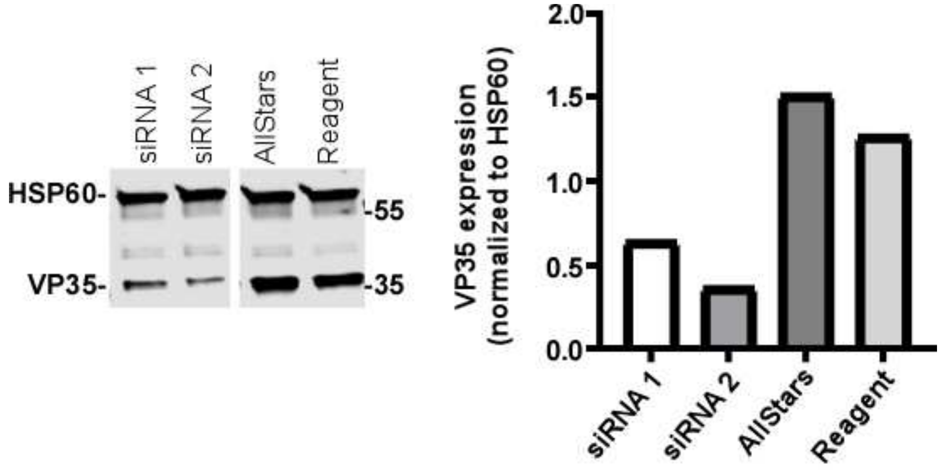

**Supplemental Figure 2: VP35 protein levels are diminished in EDC4-depleted cells infected with EBOV.** Left panel: immunoblot of lysates from EBOV challenged cells treated with EDC4 specific siRNA (siRNA 1, siRNA 2, or non-targeting siRNA (AllStars)) and stained for VP35 and loading control HSP60.

**The EDC4 C-terminal domain is responsible for recruitment of VP35 and regulates viral replication.**

EDC4 forms a scaffold on which proteins DCP1A and DCP2, the minimal proteins required for mRNA decapping[31], assemble. EDC4 contains an N-terminal WD40 domain and C-terminal proximal-distal domain, separated by a flexible linker (**Figure 5a, top**). DCP1A specifically binds EDC4 at the N-terminal WD40 domain while DCP2 binds the proximal domain at the C-terminal end[31]. The distal domain, which is highly conserved, does not bind members of the decapping complex but adopts a compact seven helix-turn-helix bundle structure and plays a role in EDC4 localization to cytoplasmic puncta associated with P-bodies in *Drosophila*[32]. To understand which part of EDC4 interacts with VP35, HA-tagged EDC4 constructs encompassing the WD40 domain plus flexible linker or flexible linker domain plus C-terminal proximal-distal region of EDC4 were transfected into HEK293T cells with FLAG-tagged VP35 (**Figure 5a, lower panel above dotted line**). VP35 strongly coimmunoprecipitated with full-length EDC4 or with the C-terminal domain but was only weakly detected with the N-terminal WD40-containing domain (**Figure 5b**). We further tested whether overexpression of C-terminal subdomains affected viral replication in a dominant negative-like manner. HA-tagged full-length EDC4 and truncations containing the C-terminal proximal-distal or distal domain alone (**Figure 5a, lower panel below**

**dotted line**) were transfected into HeLa cells, which were then challenged with EBOV. Cells were fixed at 20 hpi and viral replication was quantified via vRNAFISH (**Figure 5c**). Although expression of the proximal-distal domain demonstrated moderate effects on infection when compared to full-length EDC4 transfected samples, cells transfected with the distal domain alone showed a 6-fold reduction in infection compared to full-length EDC4 (**Figure 5c**). For each construct the frequency of transfected cells was similar though the proximal-distal domain was more highly expressed as seen by immunofluorescence (**Figure 5d**). Given that the distal domain of EDC4 plays an important role for recruitment to P-bodies, we examined how infection altered the distribution of EDC4 and DDX6, which are commonly present in P-bodies. HeLa cells were challenged with EBOV and fixed 48 hpi to allow for multiple rounds of infection. In cells with smaller VP35 inclusion body size, which represent cells in earlier stages of the EBOV infection cycle, DDX6 and EDC4 puncta were located proximally to VP35 inclusion bodies, as seen in **Figure 3c and d**. However, in cells with extensive VP35 staining, which indicates late-stage infection[33], large puncta of EDC4 and DDX6 could no longer be detected (**Figure 5e**). Overall, the data shows that VP35 interacts with the C-terminal half of EDC4, and overexpression of the distal domain disrupts infection. High level expression of VP35 seen in advanced stages of infection also caused loss of EDC4 puncta associated with DDX6, a marker of p-bodies. Further work will elucidate the importance of the EDC4 distal domain in viral infection.

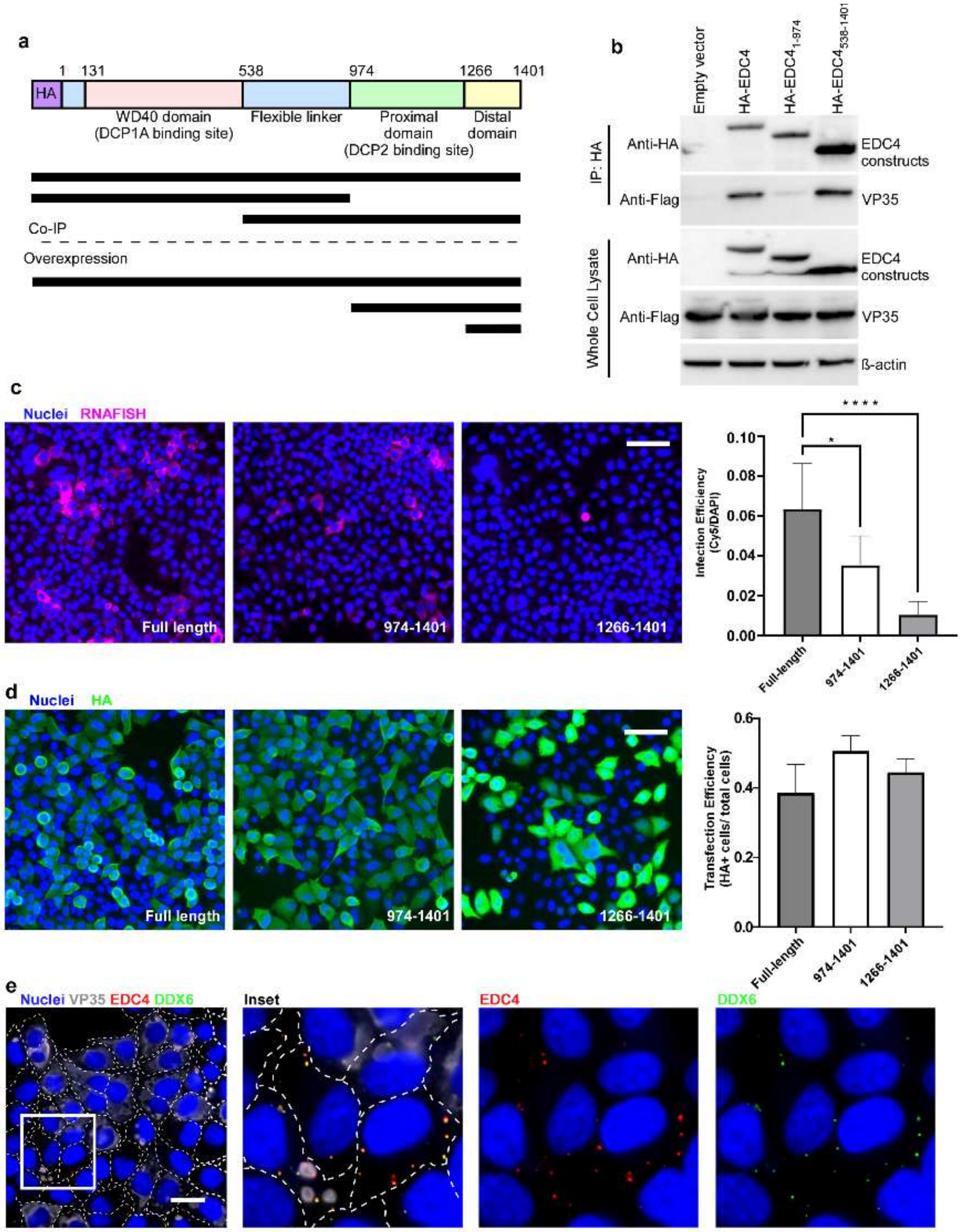

**Figure 5: EBOV VP35 interacts with the C-terminal domain of EDC4**. **a** Map of EDC4, showing binding sites for DPC1A and DCP2. Lines below the map show constructs used in co-IPs (above dashed line) and overexpression (below dashed line) experiments. **b** HA-tagged EDC4 constructs were co-expressed with FLAG-tagged VP35. VP35 did not co-precipitate with EDC4 when the C-terminal domain comprising amino acids 974-1401 was absent (lane 3). **c** EBOV infection was measured after overexpression of the indicated domain of EDC4 as determined by RNAFISH of viral mRNA (magenta) and cell nuclei stained with Hoechst 33342 (blue). *P-value >0.05; ****P-value> 0.001. **d** Transfection efficiency of EDC4 constructs as determined by HA staining (green). Scale bars = 100µM. **e** EDC4 puncta associated with DDX6. Cells were challenged with EBOV and fixed 48 hpi, then stained with antibodies against VP35 (grey) to label infected cells, EDC4 (red), and DDX6 (green). Both EDC4 and DDX6 are markers of p-bodies. Cell boundaries are indicated by dashed line. Scale bar = 25µM.

To evaluate whether EBOV replication depends on decapping components other than EDC4, we studied localization of DCP1A and DCP2 during infection and tested the impact of knockdowns of DCP2 and accessory decapping protein EDC3 on infection efficiency. Like EDC4, a portion of DCP1A and DCP2 was found in brightly stained puncta associated with VP35 stained inclusion bodies (**Figure 6a-d**, **supplemental movies 2-3**). Since DCP1A and DCP2 do not appear to directly bind VP35 **(Figure 3a**), this suggests that these components are being recruited through EDC4. Next, DCP2 and EDC3 were depleted with siRNA (**Figure 6e**) and challenged with virus. DCP2 depletion resulted in a fourteen-fold reduction of EBOV replication while EDC3 depletion resulted in a 2-fold reduction of EBOV replication (**Figure 6f-g**). Overall, our data suggests a model in which EBOV co-opts the intact decapping complex through VP35 binding to the decapping scaffold protein EDC4. Though we observed association of DCP1A and VP35, we were unable to reduce expression of DCP1A or its isoform DCP1B and excluded these proteins from further analysis.

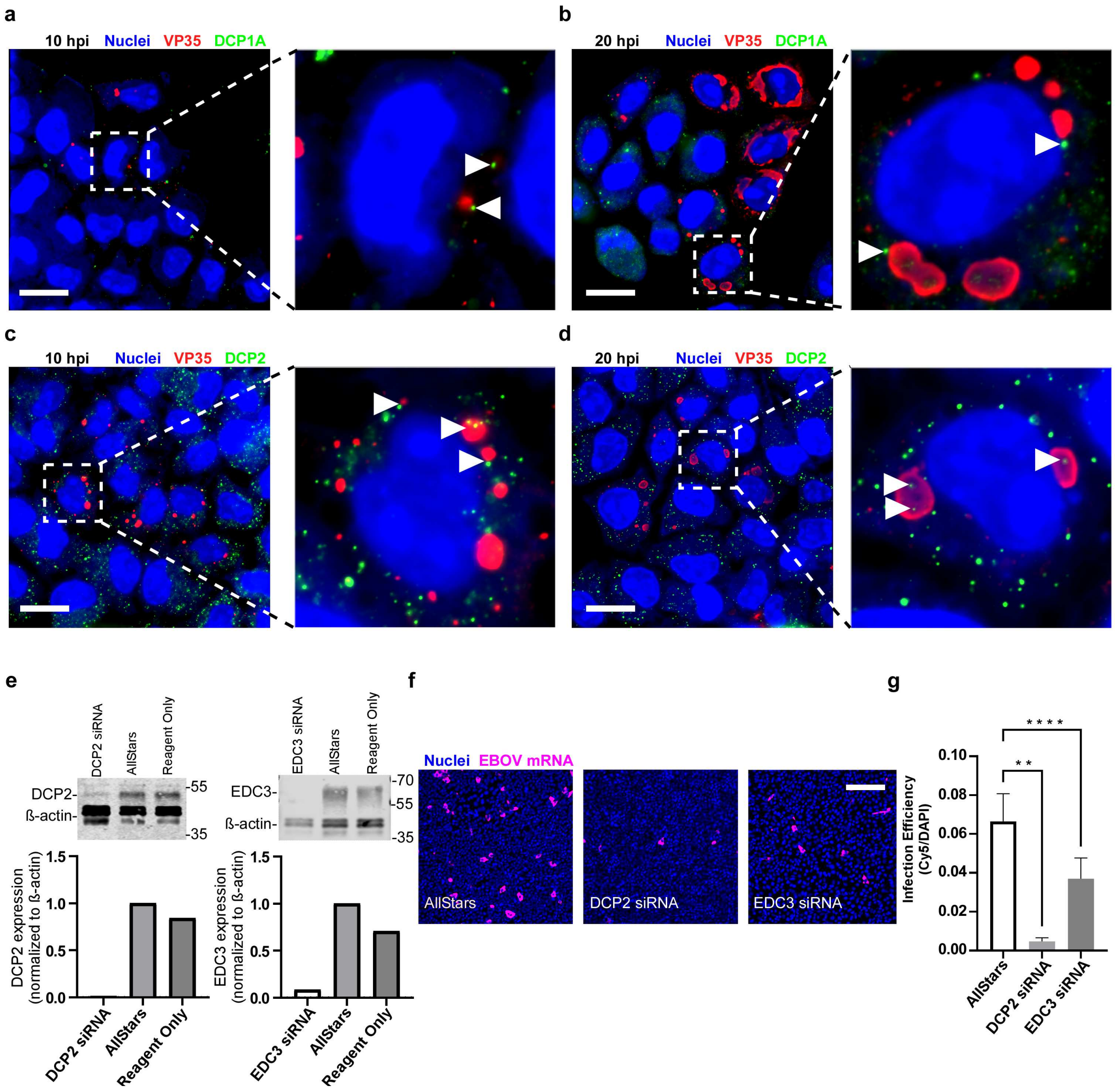

**Figure 6: EBOV replication depends on multiple components of the decapping complex. a-b** DCP1A and DCP2 colocalize with VP35 during infection. Cells were challenged with EBOV at an MOI of 1 and fixed at **a** 10 hpi and **b** 20 hpi. Proximal localization of DCP1A to VP35 inclusion bodies was observed (triangles). Scale bar = 25µM **c-d** DCP2 colocalizes with VP35 during infection. Cells were challenged with EBOV and fixed at **c** 10 hpi and **d** 20 hpi. Colocalization of DCP2 to VP35 inclusion bodies was observed (triangles). Scale bar = 25µM **e** Depletion of DCP2 and EDC3 with siRNA. **f** Impact of depletion of DCP2 or EDC3 on EBOV infection as quantitated through RNAFISH of viral mRNA. Samples were infected at an MOI of 0.8. Scale bar = 250 µM. **g** Quantification of **f** *P-value > 0.05; **P-value > 0.01; ****P-value > 0.0001.

**Discussion:**

Here we present a comprehensive protein proximity screen using all EBOV proteins except GP and define a way to inter-relate the virus-host interactome in the context of known host-host protein interactions to reveal host protein complexes. Annotating virus protein interactors onto the host protein-mapped hits, we identified members of potential host protein complexes associated with specific cellular functions, interacting with common viral proteins. Out of this analysis, we identified proteins that make up the mRNA decapping complex as important factors in EBOV replication and demonstrated that VP35 likely recruits this complex by binding to one of its members, the scaffold protein EDC4. We demonstrated that VP35 interacts with EDC4 in the same region as the enzyme DCP2, and showed that depletion of EDC4, DCP2, or EDC3 inhibited viral replication in a similar manner. In support of a direct role in viral replication, EDC4, DCP1A, and DCP2 associated with VP35-containing inclusion bodies in infected cells.

Other groups have reported interaction of isolated members of the decapping complex with filovirus proteins (**Supplemental figure 3**) but its significance was not further evaluated. Using a split-TurboID system in which an active biotin ligase was formed upon interaction of L and VP35, which together make a functional polymerase complex, Fang et al. found EDC3 in the top hits with or without VP30 while EDC4 and DCP1A were seen as secondary hits[16]. These hits were not functionally connected and other members of the complex were not identified. This may reflect the deeper coverage seen in our screen. In two independent immunoprecipitation-based screens using single virus proteins as bait, VP35 pulled down EDC4[10,34] (**Supplemental figure 3**). Again, other members of the decapping complex were not seen as partners with VP35 and the role of the interactions with EDC4 were not revealed. In our work, the role of the complex in Ebola virus replication was immediately suggested by a connected set of functionally related proteins associated by a common virus protein, VP35. This was supported by experimental verification that loss of each protein in cells gave a similar impact on viral replication. Furthermore, the proteins are seen with similar staining distributions in cells, being in puncta close to inclusion bodies containing VP35. Taken together, these data support a direct role for the entire decapping complex, not just EDC4, in regulation of viral infection.

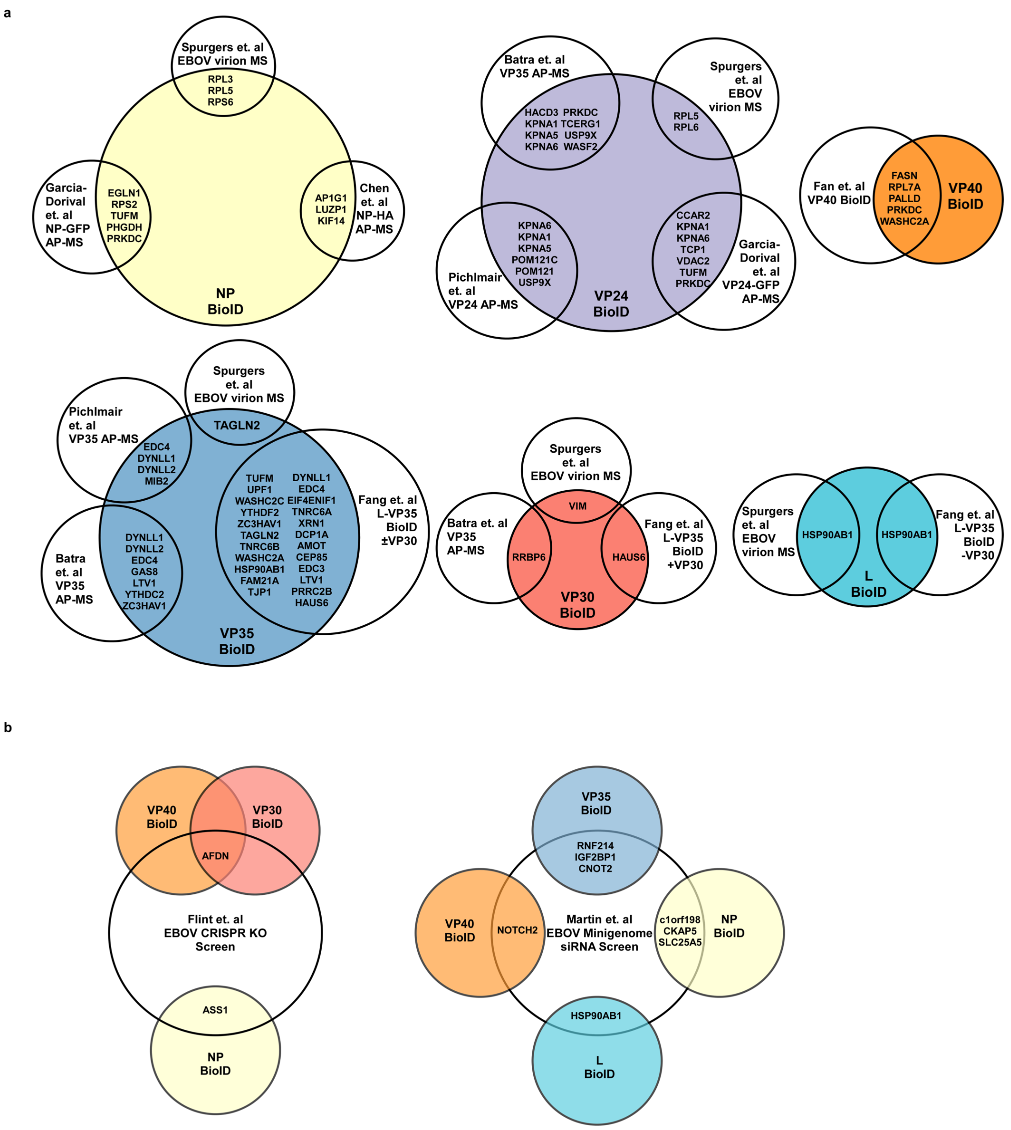

**Supplemental Figure 3:** Comparison of BioID2 screen with other EBOV PPI and genetic screens. **a** BioID hits for indicated virus proteins in this study (colored circles) were compared to other published EBOV PPI screens for those indicated proteins, including mass spectrometry of purified virus particles (Spurgers et. al[35]), affinity-pulldown mass spectrometry using NP, VP35, VP24, VP40 and VP30 as bait proteins to identify interactions (Garcia-Dorival et. al[13,14], Chen et. al[36], Pichlmair et. al[34], Batra et al[10]), and other BioID screens performed using VP40, VP35, and L to identify proximal interactors (Fan et. al[15], Fang et. al[16]). No significant overlap in hits was identified with the NP BioID screen performed by Morwitzer et. al[37]. **b** BioID hits were compared to genetic depletion screens performed for EBOV (Flint et. al[38], Martin et. al[39]). The EBOV entry screen performed by Carette et. al[40] was not included as it identified hits related to EBOV GP, which was not included in our BioID analysis.

Although the mRNA decapping complex has been implicated in the replication of other viruses[41,42], our work is the first to implicate the complex in filovirus replication and one of the few examples linking the complex to non-segmented negative sense viruses. Here we show that for EBOV, the decapping complex is required for efficient virus replication. For other viruses, components of the decapping complex exert antiviral effects. Rift Valley Fever Virus competes with host mRNA decapping enzyme DCP2 for the cellular pool of 5' methylated caps as part of its cap-snatching function[43]. XRN1, which binds EDC4 within the same region as DCP2 and degrades decapped mRNAs[31], has been implicated as both a proviral and antiviral factor for DNA and positive sense RNA viruses respectively[44,45]. In measles virus, a negative-sense virus, XRN1 promotes infection and in its absence, viral dsRNA accumulates in virus inclusion bodies[46]. Although XRN1 was labeled by VP35 in our BioID screen, our network modeling mapped it to a cluster of proteins containing UPF1 and other nonsense-mediated decay factors and so was not evaluated. The positioning of XRN1 in the network was likely due to the stringent parameters adopted for the network analysis, discussed below, and does not mean that it is absent from the decapping complex during infection. Further work is needed to determine whether XRN1 promotes EBOV infection through the decapping complex-VP35 interaction or through another RNA processing pathway.

Members of the decapping complex have been associated with processing-bodies (P-bodies). These dynamic cytoplasmic structures are composed of protein and RNA and may be involved in RNA processing and/or mRNA sequestration. P-bodies have been previously reported to fluctuate during replication of other viruses, such as SARS-CoV-2[47]. In HeLa cells, markers of P-bodies such as DDX6 and EDC4 normally formed up to six large puncta in the cell cytoplasm. However, later in infection, when VP35 demonstrated more diffuse staining in the cytoplasm, these puncta disappeared and were replaced with smaller, more numerous puncta. Though the role of P-bodies in mRNA decapping is debated[48], the C-terminal region of EDC4 that interacts with VP35 is also responsible for targeting EDC4 to P-bodies and P-body assembly[32]. Several other major components of P-bodies associated with decapping complex members were identified in our VP35 BioID screen in the network model, including DDX6, LSM14A, and LSM14B[48]. EDC3 interacts with the decapping complex by binding DCP1A and DCP1B in P-bodies[29,48]. While its role as an enhancer of mRNA decapping in yeast has been well characterized[48], its role in human mRNA decapping is understudied. Whether its labeling in our screen is due to complex interactions between DCP1A and EDC4 or is an independent interaction of VP35 requires further study.

The approach of combining proximity-dependent tagging with PPI-driven network modeling of the virus-host interactions should be readily applied to other viruses and cell types. Proximity tagging through biotinylation has advantages over pull down mass-spectrometry approaches as it can capture labile and transient complexes. However, once proteins are eluted for analysis, information on protein partners forming complexes is generally lost. Additionally, the extended set of interactions requires further down selection before starting any labor-intensive mechanistic evaluation. We rationalized that incorporation of known human protein-protein interactions into this virus-host interactome might allow recovery of protein groupings. When network tools such as STRING[20] were used to connect hits an extremely dense and complex network was produced that provided no strong guidance for follow up (**Supplemental figure 1**). Edge-density clustering methods such as MCODE[49], which predict complexes according to connection density, was applied to the STRING network and identified some putative protein complexes, but failed to incorporate many proteins that are members of well-known complexes present in screen hits (data not shown). Instead, the Prize Collecting Steiner Forest network algorithm, which seeks direct paths between hit nodes and takes into account confidence of associations from the underlying network, gave a more limited network without significant loss of mapped protein hits. We also chose to use the Human Integrated Protein–Protein Interaction rEference (HIPPIE), a well curated PPI database based on experimental evidence present in the literature[22]. The combination of both provided a greatly simplified, high stringency network that maintained cellular pathway flows. The extent of the produced network can be adjusted by tuning the stringency parameters. A balance that yielded a network of 313 members was achieved by maintaining strict edge-confidence and degree parameters in conjunction with relaxed hit incorporation. The addition of an annotated information layer of virus protein interactions and gene-set enrichment for clusters provided further insight into impact on cell pathway function and directed us to evaluate the decapping complex and its interaction with VP35. In addition, other multi-protein complexes were seen, such as those involved in nuclear-cytoplasmic transport with VP24 or microtubule polymerization with NP, that will be studied in future work.

Ultimately, our approach of screening using BioID to label neighboring proteins followed by confidence mapped network analysis helps to model the consequences of identified virus-host interactions in infection and enables prioritization of complexes for functional studies. Our work emphasizes that while most host-interaction studies readily identify 1:1 interactions it is important and helpful to identify interactions with multiple host proteins to provide mechanistic insight into how the virus is manipulating the host for its replication. This work will further help to identify major points of cellular control of EBOV infection, and better inform therapeutic development for this devastating virus and other virus types.

### **Methods:**

#### Plasmids

Plasmids myc-BioID2-MCS and MCS-BioID2-HA were gifts from Kyle Roux (Addgene plasmid # 74223; http://n2t.net/addgene:74223 ; RRID:Addgene_74223; Addgene plasmid # 74224 ; http://n2t.net/addgene:74224 ; RRID:Addgene_74224). MYC-BioID2 and BioID2-HA were PCR amplified from mycBioid2 and MCS-BIOID2-HA and inserted into pCDNA5-FRT-TO (Invitrogen) to generate plasmids pCDNA5-FRT-TO-NT-BioID2 and pCDNA5-FRT-TO-CT-BioID2. EBOV genes and GFP were PCR amplified from existing plasmids and cloned into both NT- and CT-BioID2 plasmids. All plasmids were verified by sequencing. EDC4, EDC3, DCP1a, and DCP2 plasmids were obtained from GeneScript (Piscataway NJ) and cloned into a pCAGGS expression vector with the indicated epitope tag at the N-terminus. EDC4 and VP35 truncation mutants were made from the full length pCAGGS constructs and verified by sequencing.

#### Cell lines and culture

Flp-In T-Rex 293 cells (Invitrogen, R78007) were maintained in DMEM supplemented with 10% FBS (Atlanta Biologicals Premium Select Fetal Bovine Serum, S11550) and 100 mg/mL zeocin (Gibco, R25001) at 37°C. Stable Flp-In T-Rex 293 cells containing integrated BioID2 expression constructs were grown in MEM supplemented with 10% FBS and 200 μg/ml hygromycin. All other cell lines were maintained in Dulbecco's Modified Eagle Medium (DMEM, Gibco™, Billings MT) supplemented with 10% fetal bovine serum (R&D Systems™, Minneapolis MN) at 37°C with 5% $CO_2$. HeLa cells, VeroE6 cells and HEK293T cells were purchased from ATCC® (Manassas, VA).

**Development of BioID2 stable cell lines**

Stable cell lines with the pCDNA5-BioID2 plasmids integrated were generated by co-transfecting Flp-In T-Rex 293 cells with a single BioID2 plasmid (100 ng) and either pOG44 Flp-Recombinase Expression Vector (Invitrogen, V600520, 1 μg) or pFLPo (1 μg, a gift from Philippe Soriano, Addgene plasmid # 13792; http://n2t.net/addgene:13792; RRID:Addgene_13792[50]) using Lipofectamine 2000 (Invitrogen, 11668019) according to the manufacturer's protocol. Beginning the day before transfection, cells were incubated in DMEM + 10% FBS without zeocin. On the day after transfection, the cells were transferred to a 10 cm tissue culture plate or a T75 flask. At 48 hours post transfection, the medium was replaced with DMEM + 10% FBS + 200 μg/ml hygromycin. Cells were incubated until colonies appeared (~ 14 days), at which point the cells were passaged and transferred to T75 flasks, expanded and frozen.

**Expression of the BioID2 fusion proteins.**

Flp-In T-Rex 293 cell lines containing BioID2 expression constructs were incubated in DMEM + 10% FBS + 1ug/mL tetracycline (no hygromycin) for 48 hours. Cells were dislodged from the plate by gentle pipetting with PBS, collected by centrifugation for 5 min at 500 x g, and lysed in 50mM Tris pH 8, 5mM EDTA, 150 mM NaCl, 1% Triton X-100. Protein expression was quantitated through SDS-PAGE and western blotting.

**Biotinylation of cellular proteins.**

Expression of BioID2 fusion proteins was induced by addition of tetracycline as described above. At 24 hours post addition, 50 mM biotin (Sigma, B4639) was added to the culture medium and the cells were incubated for another 24 hours, at which point cell pellets were collected. To confirm biotinylation, proteins were separated on SDS-PAGE gels and analyzed via western blotting. For mass spectrometry, cell pellets were flash frozen and stored at -80°C.

**Purification and analysis of biotinylated proteins.**

Biotinylated proteins were purified as described[51] with modifications as previously described[52]. Briefly, cell pellets from quadruplicate cultures of each BioID2 construct were thawed on ice and resuspended in 500 μl BioID lysis buffer (50 mM Tris (pH 7.5), 150 mM NaCl, 0.4% SDS, 1% IGEPAL, 1.5 mM MgCl2, 1mM EGTA) supplemented with 250 U/ml Benzonase (MilliporeSigma, 712053) and protease inhibitors (Millipore Sigma, S8830). After one freeze-thaw cycle, samples were incubated for 30 minutes at 4°C with rotation, subjected to centrifugation for 20 minutes at 21,130 x g at 4°C. Supernatants were transferred to fresh tubes containing 35 μl washed streptavidin-sepharose beads (GE Healthcare) in BioID lysis buffer. Samples were incubated overnight at 4°C with rotation. Beads were collected by centrifugation for 2 minutes at 500 x g and washed sequentially with BioID Lysis Buffer (1X), BioID Wash Buffer (50 mM Tris pH7.5, 2% SDS) (2X), BioID Lysis Buffer (2X), and 50 mM ammonium bicarbonate (pH 8.0) (3X). Beads were resuspended in 200 μl 50 mM ammonium bicarbonate (pH 8.0) and stored at -80°C after removing 10 μl to confirm pull down of biotinylated proteins by western blotting. Samples were digested with trypsin and subjected to liquid chromatography-tandem mass spectrometry (LC-MS/MS) analysis as previously described[52]. To identify biotinylated proteins, LC-MS/MS data was analyzed with Mascot Daemon with the following search parameters: Enzyme: Trypsin/P; Max missed cleavage:2; MS1 mass tolerance: 0.05 Da; MS/MS mass tolerance: 0.2 Da; Database: Human from Uniprot. Proteins identified with a false discovery rate (FDR) < 1% were submitted to analysis by SAINTexpress implemented in the Contaminant Repository for Affinity Purification (CRAPome) www.crapome.org website for analysis[19]. Proteins were considered high confidence hits if their Bayesian False Discovery Rate (BFDR) was < 0.01 when compared to negative controls (GFP-BioID2 and parental Flp-In T-Rex 293 cells incubated in with tetracycline and biotin). To identify proteins that specifically interacted with a given viral protein, mass spectrometry data from a single BioID2-tagged EBOV protein (using both NT and CT samples) was analyzed in SAINTexpress[19] using BioID data from all other viral proteins plus GFP-BioID2 and parental Flp-In T-Rex 293 cells as negative controls. For this more stringent analysis, we considered a protein to be a high confidence hit if its BFDR was < 0.05.

**Comparison to other EBOV Protein and Genetic Screens**

Using a threshold of BFDR < 0.05, an extended hit list was identified from the BioID screening. This list was compared to hit lists reported by other groups, assembled from supplementary data according to their published criteria for hit selection.

**Network Analysis and Gene Ontology Enrichment Analysis**

Because unique hits were present in both analyses described above, the resulting datasets were combined. Proteins that were identified more than once (by N- and C- terminal tagged viral proteins) were represented as one host-virus protein pairing to eliminate duplicate host-virus interactions. These proteins were then assembled into an edge list and mapped using Cytoscape 3.10.0[53].

STRING analysis was conducted using STRINGv12.0[20] with score requirements set to a confidence threshold of 0.4, and FDR stringency set to < 0.05. The resulting network was exported to Cytoscape and genes were assigned a uniform color scale.

Host protein hits identified in BioID screening were mapped onto the Human Integrated Protein-Protein Interaction rEference v2.3 using the Prize Collecting Steiner Forest Algorithm through the PCSF package[21] in R version 4.3.1. SAINT scores for hits identified in either N-terminal and C-terminal BioID screens were assigned as prize values. The optimal network solution was developed with $\omega$,$\beta$, and $\mu$ parameters set to values of 10, 2, and

0.01 respectively. 92.62% of hits reported in screening were also present in the HIPPIE interactome. Though functional enrichment was an optional application as part of the PCSF package, this portion of the package is now deprecated. To recapitulate the functional enrichment application of the PCSF package, the network resulting from application of the PCSF algorithm was clustered according to network topology using the edge betweenness community analysis algorithm in iGraph[54]. Functional enrichment analysis was performed on clusters using EnrichR through the EnrichR API package in R[23]. Functional enrichment databases were set to Gene Ontology (GO) Molecular Function 2023, GO Cellular Component 2023, GO Biological Process 2023, KEGG 2021 Human, and Reactome 2022. All R scripts written for analysis are available on Zenodo (DOI: 10.5281/zenodo.10460289), and all networks are available on NDExBio (https://www.ndexbio.org/#/networkset/bbeb3cfc-abff-11ee-8a13-005056ae23aa?accesskey=05390efc3c8e7ac5202fd1eb4cfb6e4732142df2a861be588644ef66128ee252).

**Viral Protein Network Annotation**

Host proteins identified in BioID screening were scored for interactions with each of the 6 EBOV proteins tested. If no interaction was identified between a given host protein and viral protein, a score of 0 was assigned to the host protein score. If an interaction was identified between a given host and viral protein, an initial interaction score was identified from the SAINT score reported from mass-spectrometry analysis. If a host protein was identified to interact with only 1 viral protein, the sole SAINT score was converted to a value of 1. For host proteins found to interact with multiple viral proteins, SAINT scores for all host-virus protein interactions were summed, and each individual viral protein interaction SAINT score was then rescored as a fraction of the combined saint score. Final viral protein attribute scores were assembled using the spread function in the tidyr package[55] in R and are reported in **Extended data table 3**. Scores were then assigned as network attributes using the iGraph package[54] in R, and the final attribute-mapped network was exported to Cytoscape[53] using the Rcy3 package[56] in R.

**Antibodies**

Anti-DCP1A, anti-EDC4, and anti-HA antibodies were purchased from Cell Signaling Technology (Danvers MA). Anti-DCP2 antibody was developed by Prestige Antibodies and purchased from Sigma-Aldrich (St. Louis MO). Anti-DDX6 antibody was developed by Thermofisher (PA5-18478). For immunoblotting, anti-EDC4 was purchased from Proteintech (Rosemont IL) and anti-EDC3 antibody was purchased from ABClonal (Woburn MA). Anti-β-actin antibody was purchased from R&D Systems (MAB8929). Anti EBOV VP35 antibody was developed and obtained from Dr. Daisy Leung (Washington University, St. Louis MO). Alexa-Fluor 488, Alexa-Fluor 546 goat anti-mouse and goat anti-rabbit antibodies as well as Alexa-Fluor 488 chicken anti-goat, Alexa-Fluor 546 donkey anti-rabbit and Alexa-Fluor 637 donkey anti-mouse antibodies were purchased from Invitrogen (Thermofisher Scientific, Waltham MA). Chicken anti-BioID2 primary antibody was purchased from BioFront Technologies (BioBID-CP-100).  IRDye 800CW donkey anti-chicken secondary antibody (LI-COR Biotechnology, 925-32218), IRDye® 800CW Goat anti-Rabbit IgG Secondary Antibody (LI-COR Biotechnology, 925-32211, 1/10000), and IRDye® 680RD Goat anti-Mouse IgG Secondary Antibody (LI-COR Biotechnology, 926-68070) were used as secondary antibodies for all relevant immunoblotting experiments.

**Virus propagation and infection**

All EBOV experiments were performed under biosafety-level 4 (BSL4) conditions at the National Emerging Infectious Diseases Laboratories BSL4 suite in Boston, Massachusetts USA. Zaire EBOV was originally provided by Heinz Feldmann (Rocky Mountain Laboratories, Hamilton MT) and cultured on VeroE6 cells. Supernatants were collected 5 days post infection after development of cell-pathology effect and clarified via centrifugation. Virus was then concentrated over a 20% sucrose cushion via ultracentrifugation. Titer was calculated by serially diluting the virus on HeLa cells and staining for virus via RNAFISH (see below) 20 hpi. Unless otherwise indicated, cells were infected at an MOI of 2 for all experiments. Note that treatment with transfection reagent for siRNA

and EDC4-overexpression experiments lowered the resulting cell susceptibility to EBOV.

**RNAFISH**

Samples were fixed in 10% neutral buffered formalin for at least 6 hours to inactivate EBOV. EBOV mRNA staining was adapted from previously published methods[57]. Probes targeting NP and VP35 positive-sense mRNA and containing an additional 20 nucleotide flap sequence were designed (see **Extended data table 5**) and synthesized by Integrated DNA Technologies (IDT, Coralville IA). A secondary probe complimentary to the 20-nucleotide flap sequence (see **Extended data table 5**) was synthesized with Cy5 conjugates on both 5' and 3' ends by Genewiz Inc. (Azenta Life Sciences, Burlington MA).

**Immunofluorescence and Proximity Ligation Analysis**

Samples were fixed in 4% paraformaldehyde for at least 6 hours to inactivate EBOV. Samples were then washed in phosphate-buffered saline (PBS) and permeabilized in 62.5 ng/mL digitonin, then blocked in 3.5% bovine serum albumin (BSA). Primary antibodies were diluted in 3.5% BSA and incubated at 4°C overnight. Samples were washed in PBS, and secondary antibodies were diluted in 3.5% BSA and incubated for 1 hour at room temperature. Cell nuclei were stained with Hoechst 33342 (Thermofisher Scientific, Waltham MA) diluted in PBS. PLA was performed using the EDC4 and VP35 antibodies described above with the Duolink In Situ Detection Red Kit (Millipore Sigma, Burlington MA) according to the manufacturer's protocol.

**Co-immunoprecipitations**

HEK293T cells in 6 well plates were transfected with the indicated plasmids and amounts (HA-DCP1a 500ng, HA-DCP1b 500ng, HA-DCP2 500ng, HA-EDC3 500ng, HA-EDC4 500ng, HA-EDC4 1-974, HA-EDC4 538-1404, Flag-VP35 1ug) by using Lipofectamine 2000 according to the manufacturer's instructions (Invitrogen #11668500). After 48 h incubation, the cells were washed once with PBS, lysed in 240µl NP40 lysis buffer (50mM Tris HCl pH 8, 280mM NaCl, 0.2 mM EDTA, 2mM EGTA 10% glycerol, 0.5% NP40) and centrifuged at 15000 rpm for 10 min at 4°C. The whole cell extracts were incubated with Anti HA magnetic beads (Pierce™ Anti-HA Magnetic Beads #88836) or anti FLAG M2 beads (Sigma-Aldrich) for 45 minutes. A small portion of supernatant (40µl) was saved as input. The beads were washed five times with NP40 lysis buffer. The IP complex was eluted in 50µl NP40 buffer containing 1X SDS loading buffer (Thermo- scientific #J61337-AC) and heated to 95°C for 5 minutes. Proteins were separated by SDS-PAGE gels (Invitrogen™ Bolt™ 4 to 12%, Bis-Tris #NW04120BOX) and subsequently transferred onto a PVDF membrane (Sigma-Aldrich #03010040001). The membranes were blocked with 5% blocking buffer (#1706404) for 1 h at room temperature followed by overnight incubation with anti-Flag (Sigma-Aldrich #F7425) and anti-HA (Invitrogen #71-5500) antibodies. The membranes were then incubated with secondary antibody (Cell Signaling Technology #7074S), washed for 3 times, 10 min each using 1X PBST and finally imaged with Western Chemiluminescent HRP substrate (PerkinElmer #NEL104001EA) in a ChemiDoc™ MP Imaging System (Bio-Rad).

**siRNA and Plasmid Transfections**

Scramble control siRNA and siRNAs targeting EDC4, EDC3, and DCP2 were purchased from Qiagen (Hilden, Germany). HeLa cells were seeded at 6000 cells per well in 96-well dishes. siRNAs were transfected at a concentrations of 5nM (EDC4 siRNA) or 10nM (EDC3 and DCP2) siRNA per 96 well using 0.3µL of Lipofectamine RNAiMax transfection reagent (Thermofisher Scientific, Waltham MA). 24 hours post transfection, cells were washed in growth media and infected with Zaire EBOV. For EDC4 expression experiments, full-length and truncated EDC4 plasmids were transfected into HeLa cells at 0.1µg per 96 well using TransIT-LT1 (MirusBio, Madison WI) according to the manufacturer's protocol. 24 hours post transfection, cells were challenged with Zaire-EBOV.

**Microscopy and Image Quantification**

For EDC4 knockdown experiments in which RNAFISH was used as a metric of viral replication efficiency as well as EDC4 truncation expression experiments, plates were imaged on a BioTek Cytation 1 (Agilent Technologies, Lexington MA) using a 10x objective and Cy5 and DAPI filters. Each well was imaged by collecting 16 nonoverlapping tiled images. For DCP2 and EDC4 KD experiments, plates were imaged on a Nikon Ti2 fluorescent microscope (Nikon, Tokyo Japan) using a 10x objective and 385 and 637 filters. For all decapping component KDs, infection efficiency was quantified by measuring the area occupied in $\mu m^2$ by 637 and 385 signals corresponding to RNAFISH and cell nuclei in ImageJ or CellProfiler[58]. RNAFISH area occupied and nuclei area occupied measurements summed for each well and total RNAFISH values were normalized to total nuclei area occupied measurements to generate infection efficiency. For infection experiments examining EDC4 and DDX6 distribution, images were taken on a Nikon Ti2 fluorescent microscope (Nikon, Tokyo Japan) using a 20x objective and 385, 490, 550, and 637 filters. For PLA experiments, Z-stack images were taken using a 100x oil-immersion objective with 2µM separating each z-slice and 385 and 637 filters. Images were deconvolved using Microvolution (Microvolution LLC, Cupterino CA) in ImageJ and processed to give maximum intensity projections. For colocalization experiments between VP35 and decapping components, images were modeled using Imaris (Oxford Instruments, Concord MA). VP35 and decapping component signals were thresholded, with VP35 modeled as a surface and decapping component punctae modeled as spots.

**SDS-PAGE and Western Blotting**

To analyze BioID2 fusion protein expression, samples were lysed as described above and were combined with 2X SDS sample buffer and boiled, and proteins were separated on an SDS-PAGE gel. Samples were transferred to nitrocellulose and sequentially probed with primary and secondary antibodies. To confirm biotinylation in BioID screens, samples were lysed as described above and transferred to nitrocellulose and probed with IRDye 800CW Streptavidin (LI-COR Biotechnology,926-32230). For mRNA decapping component KD and viral protein quantitation, Samples were lysed with RIPA buffer supplemented with Pierce Protease and Phosphatase Inhibitor (Thermofisher Scientific, Waltham MA) and boiled in 1% SDS at 100°C for 10 minutes to denature samples and inactivate EBOV. Samples were clarified via centrifugation, then combined with laemmli and loaded onto 4-20% polyacrylamide Mini-PROTEAN® TGX gels (BioRad, Hercules CA). Following electrophoresis, samples were rapidly transferred to nitrocellulose membranes using the Trans-Blot Turbo transfer system (BioRad, Hercules CA) and blocked in Intercept (PBS) blocking buffer (LI-COR Biosciences, Lincoln NE) at room temperature for 1 hour with rocking. Antibodies were diluted in Tris-buffered saline-tween (TBST) and membranes were incubated overnight at 4$^{o}$C with rocking. Membranes were washed twice in TBST, then incubated with fluorescent secondary antibodies diluted in blocking buffer for 1 hour. Membranes were then washed three times in TBST. Target protein quantities were normalized to housekeeping protein quantities. All blots were imaged on LI-COR Odyssey CLx or LI-COR Odyssey Imaging Systems and quantified in ImageStudio Lite (LI-COR Biosciences, Lincoln NE).

**PCR, Reverse-transcription PCR (RT-PCR), and Quantitative PCR (qPCR)**

All decapping component KD samples infected with EBOV were lysed in TRIzol Reagent (Thermofisher Scientific, Waltham MA), and RNA extractions were performed via chloroform precipitation. RT-PCR was performed using the PrimeScript RT Reagent Kit (Takara Bio Inc., Kusatsu, Shiga, Japan), using oligo-DT primers to amplify mRNA, random hexamers to amplify non-capped and non-poly-adenylated RNAs, or EBOV-genomic specific primers. Quantitative PCR detecting viral mRNA was performed using the Luna Universal Probe qPCR Master Mix (New England Biolabs Inc, Ipswich MA). Quantitative PCR detecting antigenomic and genomic EBOV strands was performed using the iTaq Universal SYBR Green Supermix (BioRad, Hercules CA). Primers and probes used to detect viral mRNA were designed using the IDT PrimerQuest design tool for PrimeTime qPCR assays. NP primer and probe sequences are as follows: 5'- ACTCCATCACGCTTCTTGAC-3' (forward primer), 5'-CATGCGTACCAGGGAGATTAC-3' (reverse primer) and 5'-/56-FAM/TCAAGTATT/ZEN/TGGAAGGGCACGGGT/IABkFQ/-3' (probe). GP primer and probe sequences are as follows: 5'-CAGTCCGGTCCCAGAATGTG-3' (forward primer), 5'-TTTTCAATCCTCAACCGTAAGGC-3' (reverse primer), and 5'-/56-FAM/-CATGTGCCG/ZEN/CCCCATCGCTGC/IABkFQ/-3' (probe). 7SK snRNA was used as a housekeeping gene, and was quantitated using assay Hs.PT.58.38798776.g from IDT. Primers to detect EBOV genomic and antigenomic strands have been previously described[30].

**Statistical Analysis**

GraphPad Prism 10 for Windows (GraphPad Software, San Diego CA) was used to performed one-way ANOVAs. Significance was determined as any p-value < 0.05. All experiments were performed with at least 3 independent replicates, with at least three technical replicates per independent experiment.

### Data Availability

All raw LC-MS/MS data files are deposited in MassIVE data repository (https://massive.ucsd.edu/) with the dataset ID MSV000093669. All R scripts written for analysis and CellProfiler pipelines are available on Zenodo (DOI: 10.5281/zenodo.10460289), and the final network map is available on NDEx (https://www.ndexbio.org/#/networkset/bbeb3cfc-abff-11ee-8a13-005056ae23aa?accesskey=05390efc3c8e7ac5202fd1eb4cfb6e4732142df2a861be588644ef66128ee252).

### Acknowledgements

All the LC-MS/MS experiments including data analysis were performed at the Purdue Proteomics Facility, Bindley Bioscience Center, Purdue University. The authors would also like to thank Jacquelyn Turcinovic at Boston University for assistance with R script writing. Funding support was provided by NIH/NIAID grants R01AI114814 and P01AI120943.

## Supplementary Files

This is a list of supplementary files associated with this preprint. Click to download.

- ExtendedDataTables.zip
- DecapVP35Movies.zip
- WemadetheMassspecdataisaccessiblethroughthislink.docx
- DonahueDaveynrsoftwarepolicy.pdf
- nrreportingsummaryDonahueetal.pdf
